# Ambient Agents: Embedded Agents for Remote Control and Monitoring Using the PANGEA Platform

**DOI:** 10.3390/s140813955

**Published:** 2014-07-31

**Authors:** Gabriel Villarrubia, Juan F. De Paz, Javier Bajo, Juan M. Corchado

**Affiliations:** 1 Departamento de Informática y Automática, Universidad de Salamanca, Plaza de la Merced s/n, 37008 Salamanca, Spain; E-Mails: gvg@usal.es (G.V.); corchado@usal.es (J.M.C.); 2 Departamento de Inteligencia Artificial, Universidad Politécnica de Madrid, Campus de Montegancedo s/n, Boadilla del Monte, 28600 Madrid, Spain; E-Mail: jbajo@fi.upm.es

**Keywords:** ambient intelligence, smart living, remote monitoring, open MAS

## Abstract

Ambient intelligence has advanced significantly during the last few years. The incorporation of image processing and artificial intelligence techniques have opened the possibility for such aspects as pattern recognition, thus allowing for a better adaptation of these systems. This study presents a new model of an embedded agent especially designed to be implemented in sensing devices with resource constraints. This new model of an agent is integrated within the PANGEA (Platform for the Automatic Construction of Organiztions of Intelligent Agents) platform, an organizational-based platform, defining a new sensor role in the system and aimed at providing contextual information and interacting with the environment. A case study was developed over the PANGEA platform and designed using different agents and sensors responsible for providing user support at home in the event of incidents or emergencies. The system presented in the case study incorporates agents in Arduino hardware devices with recognition modules and illuminated bands; it also incorporates IP cameras programmed for automatic tracking, which can connect remotely in the event of emergencies. The user wears a bracelet, which contains a simple vibration sensor that can receive notifications about the emergency situation.

## 1. Introduction

Recent years have seen great advances in the field of ambient intelligence, which has assumed significant importance in the daily lives of individuals. Ambient intelligence tries to adapt technology to people's needs by proposing three basic concepts: ubiquitous computing; ubiquitous communication; and intelligent user interfaces [1]. In order to reach this objective, it is necessary to develop new frameworks and models to allow access to functionalities regardless of time and location restrictions. People are currently surrounded by technology that tries to increase their quality of life and facilitate daily activities. In addition, continuous advancements in mobile computing make it possible to obtain contextual information and to react physically to the context in more innovative ways [2]. These advances have led to mobile devices or sensor networks with limited processing and storage capacities that are used to design ambient intelligence environments. These devices are used within an intelligent environment in order to obtain information or to respond to the environment and, in the majority of cases, interact with the rest of the intelligent system [3].

Wireless sensor networks (WSN) provide an infrastructure capable of supporting the distributed communication needed in highly dynamic environments, increasing mobility, flexibility and efficiency, since resources can be accessed regardless of their physical location [4]. Sensor networks interconnect a large number of sensors and manage information gathered from intelligent environments. On many occasions, information management is distributed; however, it is necessary to have distributed systems sufficiently capable of managing WSN effectively, and that includes software elements containing a certain degree of intelligence that can be embedded into devices and respond both autonomously and in conjunction with the distributed system. Multi-agent systems are a very appropriate option for this type of system. There are different proposals that combine multi-agent systems and sensor networks to build intelligent environments [5–10] in ambient intelligence. However, in the field of ambient intelligence, there is currently no multi-agent architecture that functions on the concept of virtual organizations and that can provide agents capable of being embedded in devices.

Recent trends in the development of multi-agent systems to manage wireless sensor networks are based on social computing and virtual organizations. Given the special characteristics of multi-agent systems, multi-agent architectures have been explored during recent years as an emerging trend to manage wireless sensor networks [11–14]. This new perspective can provide an organizational management model appropriate to manage both internal behaviors and user services more efficiently. In this sense, social computing and organizational aspects can notably help to design new platforms with advanced capacities to interconnect and manage WSNs. In this article, we focus on the interaction with the environment, designing intelligent agents that can be embedded into hardware resource-constrained devices. This article presents the integration of the PANGEA (Platform for the Automatic Construction of Organiztions of Intelligent Agents) platform with different hardware to develop a context-aware system. An agent is embedded in an Arduino microcontroller [15]. A device without processing capacity is also integrated; for this case, we have selected an Internet Protocol (IP) camera. The deployed agents are managed through the PANGEA [16] platform, a multi-agent architecture designed on the base of a virtual organization and oriented toward the creation of ambient intelligence environments. PANGEA allows for easy integration with different hardware and several languages, due to the simplicity of the communication protocol, which is based on Internet Relay Chat (IRC). This makes it easy to find libraries in different languages or to develop specific libraries according to the IRC standard. PANGEA is a multi-agent architecture platform aimed at creating open multi-agent systems that provide various tools to create, manage and control virtual organizations. PANGEA is specifically designed to develop multi-agent systems that include organizational aspects. Two innovations presented in PANGEA are the sensor agent and the vibrator agent, which are embedded in lightweight devices that obtain context-aware information and manage actuators. The sensor agent and vibrator agent are autonomous agents embedded in an Arduino microcontroller, integrated within the PANGEA platform, which provides several advantages to manage context-aware information. In addition to the sensor agent and vibrator agent, the system also presents the camera agent, which controls the IP cameras that respond automatically to movement. The sensor agent is used to detect several kinds of alarms that are installed in a laboratory. According to the detected sounds, the agent engages a specific action in accordance with the established rules. Although the alarms are not connected to the WSNs, the sound detector created by the sensor agent can detect the sounds and respond accordingly. PANGEA allows the integration of mobile devices, facilitates a new ubiquitous communication process and provides a new mechanism to incorporate intelligent behaviors into embedded devices. These characteristics are relevant to develop intelligent environments.

The article is structured as follows: We begin with a review of the state-of-the-art of agents and multi-agent systems, with particular attention to agents that are used as embedded software. The next section describes the PANGEA platform, providing the detailed structure of the sensor agent, camera agent and vibrator agent. The architecture, as well as the sensor agent and camera agent are evaluated in a case study using an intelligent environment especially designed for indoor fire detection. The final section presents the results obtained from the case study and the conclusions that were drawn from this study.

## 2. Related Work

Ambient intelligence is a new paradigm that tries to adapt technology to the needs people have. The emergence of ambient intelligence involves substantial changes in the design of functional architectures, since it is necessary to provide features that enable ubiquitous computing and communication and also an intelligent interaction with users. The work of Acampora and Loia [17] highlights that ambient intelligence systems incorporate the following features: context awareness, multimodal communication and user-centered interaction. This section revises the state-of-the-art of related developments, which integrate WSNs, mainly based on multi-agent systems, focusing on the possibility of using autonomous, embedded agents, and analyzes the feasibility of a new alternative using the PANGEA platform.

Context-aware systems were first introduced by Want *et al.* [18] when they presented their Active Badge Location System, generally regarded as the first context-aware application. It is a location system for individuals in an office environment in which each person carries a badge that uses a sensor network to send signals containing information about each person's location to a centralized services area. By 2005 and through the end of the decade, several tourist guide location-aware systems emerged [19,20], which provided information about the user's location. Location information is by far the most used attribute of ambient intelligence systems. Recent years have seen considerable growth in the use of other attributes of context-aware information. One of the most precise definitions was provided by Dey and Abowd [21]. These authors refer to context as any information that can be used to characterize the situation of an entity. An entity is a person, place or object that is considered relevant to the interaction between a user and an application, including the user and the applications themselves.

Developments are currently underway to create applications that allow integrating sensor networks. These developments are based, for example, on the TinyOS operating system, which allows for the interconnection of sensors. Its main characteristic is low power consumption. The operating system can be used on systems, such as Mica2, and is widely used in research [22]. Many projects have been developed over this operating system, including Maté, which is a virtual machine that can interpret bytecodes [23]. Maté has also been used to develop other agents, such as the Agila middleware [24], which can create agents with mobility functions; it has also been used in fire tracking. A study by Mlla *et al.* [25] shows some existing middleware, some of which has been created over TinyOS. It is also possible to find simulators for TinyOS, such as TOSSIM [26], which facilitate the development of this kind of application. Although TinyOS allows remote communication with other devices, it does not incorporate characteristics to facilitate the development of distributed applications to easily manage the services provided by the agents and control and monitor the interactions among the agents in the platform.

Multi-agent architectures have been explored as an alternative to develop context-aware systems. Lim *et al.* [27] propose a project to create a context-aware home that utilizes multi-agent systems to monitor and execute appropriate actions based on the current state of the house. The multi-agent system learns and adapts the movements and actions of the occupants and makes predictions. The authors propose an architecture that includes an agent in each room that interacts with the sensors and a superagent that makes decisions and deals with risk prediction. Other authors, such as Uhm *et al.* [28], focus on the semantic aspects of the context and propose a context model that separates the upper and lower layers according to the characteristics of each class using the Web Ontology Language (OWL). The system does not provide either embedded agents or organizational aspects. Kaluza *et al.* [29] present a context-aware multi-agent system for the care of the elderly that combines sensor technologies to detect falls and other health problems, and either calls for help in the case of an emergency or issues a warning in cases that do not require immediate attention. The ambient intelligence system focuses on detecting alarm situations and does not provide embedded agents or organizational aspects. Some studies, such as the one proposed by Ning and Yang [30], present agents embedded in agents to control their functioning. Doctor *et al.* [31] use embedded agents in mobile devices and fuzzy logic to emulate certain human behaviors [32].

Some existing platforms, such as JADE (Java Agent Development Framework) [33,34] and AFME (Agent Factory Micro Edition) [35], allow the execution of agents in mobile devices, but require the use of specific libraries that are only available for certain platforms, such as Android or J2ME (Java 2 Micro Edition). JaCa-Android [35] facilitates the execution of intelligent agents in Android devices. MAPNET [36] is focused on mobility and allows the creation of mobile agents that can be executed in different platforms using the MASIF (OMG Mobile Agent System Interoperability Facility) standard [37]. As with MAPNET, UMAP (Universal Multi-Agent Platfom for .NET) [38] is based on .Net and requires Microsoft programming languages. ICARO-D project [39] provides RMI (Remote Method Invocation) communications to distribute resources among agents. In robotics, there have been different agents embedded in robots [40], but without platform support. Purusothaman *et al.* [41] show an alternative to creating multi-agent systems in Arduino devices. The platform deployed in the Arduino devices only contains the basic functionality to be executed in these devices. This alternative allows reducing costs, although there are systems on a chip, such as Raspberry Pi [42], with higher computational capacity and a similar price; although the energy consumption is greater.

Agent-based virtual organizations can provide new capacities to create artificial societies. This area has grown during recent years with most studies focused on security [43], planning [44], role assignment [45], resource management [46], collaboration [47], *etc*. However, it is not possible to find approaches based on virtual organizations of agents and ambient intelligence, where agents can be embedded in resource-constrained devices. It is possible to find platforms especially designed to manage virtual organizations of agents, such as Janus [48], that allow the instantiation of agents in Android devices. MaDKit (Multiagent Development Kit) [49] is similar to JADE. Other platforms such as CONOISE-G [50], are founded on JADE, but provide support for virtual organizations of agents.

Previous work by the authors of this paper [51] allows the installation of embedded agents, but the proposed HERA (Hardware Embedded Reactive Agents) platform allows only the execution of reactive agents and does not provide support for virtual organizations. Moreover, the platform is based on a service-oriented architecture, which notably complicates communication with hardware devices, such as Arduino. The THOMAS (MeTHods, Techniques and Tools for Open Multi-Agent Systems) platform [31] is designed to create and manage virtual organizations of agents, but does not allow the creation of agents from embedded devices. Virtual organizations would provide artificial intelligence systems with greater dynamism, allowing services to be modified in execution time, defining interaction norms, monitoring traffic, *etc*. In addition, virtual organizations will facilitate the management of multiple WSNs by a single platform by creating different virtual organizations for each WSN.

The goal of the present study is to move one step further toward the development of an organizational-based architecture that incorporates embedded agents to store and process different types of data gathered by the system in order to improve the performance of ambient intelligence environments.

As shown in this section, ambient intelligence requires novel solutions to develop ambient intelligence environments and, more specifically, to manage context-aware information. Multi-agent systems and WSNs are essential technologies for this aim. However, it is still necessary to design effective architectures incorporating embedded agents. The next section presents the multi-agent architecture proposed in this paper, focusing on the sensor agent, which incorporates an innovative embedded agent model.

In addition to the sensor agent, this study also presents the camera agent. This agent is a common agent that is not embedded in the hardware and whose intelligent behavior is executed on the hardware devices. The new communication between these agents is established through a new protocol, which provides an added value for developing ambient intelligence environments.

## 3. Proposed System

The proposed system is developed over the PANGEA platform. PANGEA integrates different agents that control the operation of the devices connected to the system. In Sections 3.1 and 3.2, the platform is introduced with references to include more detail. PANGEA facilitates embedding agents in low performance devices. The agents that compose the different virtual organizations (VO) in Arduino devices are explained in Section 3.3. These agents allow the devices to recognize and treat different sounds. Finally, Section 3.4 includes information about the agent in charge of the camera.

### 3.1. Overview of PANGEA

With the evolution of ubiquitous and distributed systems, it is necessary to create new agent platforms that facilitate the development of open agent architectures and ambient intelligence systems [52], which must be capable of deploying their agents in any device. PANGEA [16] is an agent platform based on organizational concepts that can model and implement all kinds of open systems, encouraging the sharing of resources and facilitating the control of all nodes where the different agents are deployed.

One of the main features of PANGEA, which provides great flexibility to developed systems, is that it is geared towards VO agents [53]. A VO must be understood as an open system formed by the grouping and cooperation of heterogeneous entities and where there is a gap between the form and the function that defines its behavior [54].

The main features that a VO must support are:

- Creating organizations and sub-organizations: These organizations largely determine the communication and interaction capabilities among the agents. Moreover, each organization is determined by a structure or topology that can be altered throughout the lifecycle of the organization.
- Management of roles: Agents within the organizations are mainly characterized by the role they play, which determines their abilities, skills or services.
- Management of services: Agent skills are understood as services. Thus, providers and requester agents emulate a client-server architecture, where agents offer their services and other agents demand such services.
- Rules: One of the main reasons for the creation of the VOs was the need to emulate social systems (hence, the need for a structure). Rules are also part of social systems; in highly dynamic environments, control mechanisms that facilitate the integration of heterogeneous components must be established. This role is played by the rules within VOs.

The VOs can be considered an evolution of the multi-agent system (MAS), where new features come into play that allow systems to be more open and, thus, more flexible and adaptable. With these new features, the number of platforms available for the development of open systems is reduced, hence the need for the development of the PANGEA platform.

Some of the features that make PANGEA a highly recommended platform for AmI systems are: (i) the ability to include heterogeneous agents in terms of languages and runtime platforms; (ii) a robust and reliable communication mechanism; and (iii) the ease of implementation.

This platform has been used to develop many systems in fields related to AmI, such as information fusion [55] or personalization of the workspace by a proximity detection system [56]. More detailed information about the platform is already published and can be consulted in [57,58].

### 3.2. Integration of the Organizations in PANGEA

**Figure 1.**
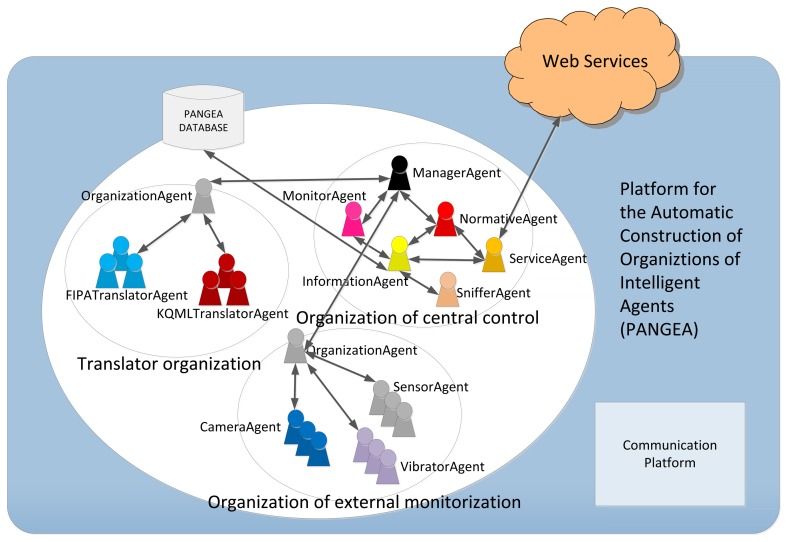
Overview of the agents deployed in the PANGEA platform. FIPA, Foundation for Intelligent Physical Agents; KQML, Knowledge Query and Manipulation Language.

The PANGEA platform is a general purpose platform that can be used to implement different applications based on VOs. However, due to the fact that monitoring and control mechanisms common to all fields of study are needed, PANGEA automatically displays an organization called the Central Control Organization. As explained in detail below, all of the agents belong to this organization in order to ensure the correct operation of the platform and the life cycle of agents, the control of standards and the allocation of roles, among other factors. Moreover, specific organizations were included for this case, allowing the tasks of the described system to be carried out. Figure 1 shows the agents included in each organization.

The organizations are:

- Translator Organization: The agents deployed in this organization are in charge of the translation and communication with agents that do not share the same Agent Communication Language (ACL) used by the PANGEA agents. PANGEA agents are implemented in Java or C++ and use the standard Internet Relay Chat (IRC) as the communication language. Currently, this organization includes agents with translation capabilities for communication languages, such as Knowledge Query and Manipulation Language (KQML) or FIPA-ACL (Foundation for Intelligent Physical Agents) [59].
- Central Control Organization: this organization includes the agents that manage and control the entire platform, which will be seen in the next section.
- Organization for External Monitoring: This organization includes those agents deployed in external monitoring devices, such as cameras, sirens, *etc*. These agents will be explained in detail in subsequent sections.

As mentioned previously, VOs should offer organizational norms, roles and the services associated with them. The agents that ensure these features in PANGEA are deployed in the Central Control Organization. Agents that are part of PANGEA can be executed on different computers, based on a decentralized scheme. A new agent may access the platform specifying the IP address of the machine on which the ManagerAgent agent is deployed. This agent is responsible for ensuring efficient and secure communication. Other agents in the system can be executed or replicated on other machines, ensuring the recovery of a possible fault in a network node.

- OrganizationManager: responsible for the actual management of organizations and suborganizations. It is responsible for verifying the entry and exit of agents and for assigning roles. To carry out these tasks, it works with the OrganizationAgent, which is a specialized version of this agent.
- InformationAgent: responsible for accessing the database containing all pertinent system information.
- ServiceAgent: responsible for recording and controlling the operation of services offered by the agents. It works as the Directory Facilitator defined in the FIPA standard.
- NormAgent: ensures compliance with all of the defined norms in the organization. This agent is responsible for ensuring that all communication complies with established restrictions and that only certain agents communicate with other specific agents. This functionality will provide security in the platform.
- Sniffer: manages the message history and filters information by controlling communication initiated by queries.
- MonitorAgent: controls the life cycle of other agents and enables the interface to display the general state of the communications, organizations and agents. This agent is responsible for starting the agents of the platform in case of failure.

The PANGEA platform is executed one time only for different WSNs; in other words, an organization that manages the elements of the WSN is created for each case study. The External Monitoring Organization was created specifically for this case study.

The main feature of the Organization for External Monitoring is to contain all of the agents who are responsible for managing and controlling the agents deployed in external devices and the agents responsible for controlling devices, in this case, the SensorAgent, CameraAgent and VibratorAgent.

An important aspect is that the platform can limit unwanted communications among different organizations. Thus, an agent from the External Monitoring Organization could not communicate with another agent from another organization unless it is enabled by a norm in the NormAgent. The agent platform is therefore unique and can be reused in different case studies. In addition, all agents, except the ManagerAgent, can be replicated in the system; therefore, if the system grows, a new agent can be deployed in another node.

### 3.3. Sensor Agent

The main goal of this section is to describe the functionalities of the sensor agent model that was developed and to mention its primary advantages as compared to the systems traditionally used to deploy agents. Embedded systems are designed to perform very dedicated or simple functions. Their primary use is for real-time computing applications. One of the most notable characteristics of using embedded software, with regard to other computing systems, is the low manufacturing cost, which is a result of the small size of the processor and memory in the device. There are different microcontrollers and systems on a chip currently available in the market, the most well-known microcontrollers being Arduino [60] and Propeller [61], and the most well-known systems on a chip being Beagle Board [62] and Raspberry Pi [42]. The Arduino platform was selected for the development of embedded agents in the present study, due primarily to the following advantages: it is more economical; it uses a simple multiplatform, with scalability-hardware and scalability-software.

Although the aim of this work is to define a general sensor agent model that could be embedded in different devices, in order to show the effectiveness and the potential of the proposal, we have focused on the Arduino devices and some specific functionalities, all of which will be further developed in future studies. The sensor agent model presented in this paper is defined by means of a recognition model aimed at detecting sounds, an actuator system based on light control and a communication module with the PANGEA architecture. The communication is established through the IRC protocol in such a way that each of the sensor agents can communicate with the corresponding coordinator of an organizational unit of the organization. One of the advantages of the proposed sensor agent model is that each sensor agent embedded in a device has a corresponding sensor agent executed in the PANGEA platform. This way, the sensor agent in PANGEA can execute complex processing tasks that cannot be carried out by the embedded agent due to resource constraints. The sensor agent will execute a sound recognition algorithm in those cases where the embedded agent cannot classify the sound. In the following paragraphs, we define each of these components.

#### 3.3.1. Recognition Module

The proposed sensor agent incorporates a recognition module. The processing and subsequent acoustic analysis is done with an EasyVR board [63], which provides simple connectivity with any controller through Universal Asynchronous Receiver-Transmitter (UART) [64] communication. The sounds to be recognized are stored in the internal memory, with a capacity of up to 32 sounds. It includes a microphone that allows for the continuous capture of sounds in the environment and consequently generates a response.

**Figure 2.**
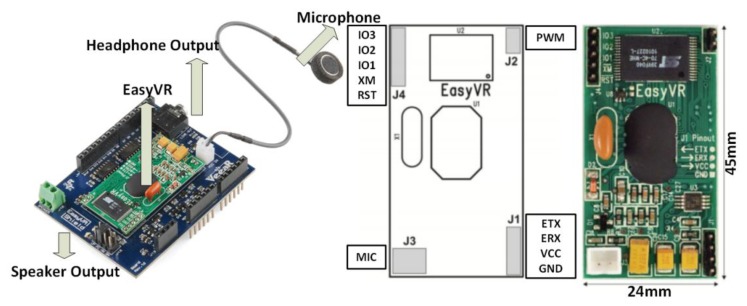
The voice recognition module for Arduino.

The minimum power supply needed to work with this module is 3.3 V, which is sufficient to work together with the Arduino Duemilanove board. Figure 2 shows a diagram of the module.

When the embedded agent cannot classify a sound, the sound is sent to the corresponding replicated sensor agent in the PANGEA architecture to execute a more precise classification algorithm. This allows the system to avoid sending data continuously, thereby sending information only when an unknown sound is detected. The replicated agent is used to carry out tasks with high processing. The agent in the Arduino device sends the information to the replicated agent with the service analysis to conduct this processing. The replicated agent implements a sound recognition algorithm that combines the Shazam algorithm [65], a very robust method to distinguish noise, with the Mel frequency cepstral coefficients (MFCC) technique [66,67]. MFCC and Shazam work in a similar way, obtaining a fingerprint of the sound; MFCC uses spectrogram peaks, while Shazam uses local maximums in the signal frequency curve.

#### 3.3.2. Sensor Agent Functionalities

**Figure 3.**
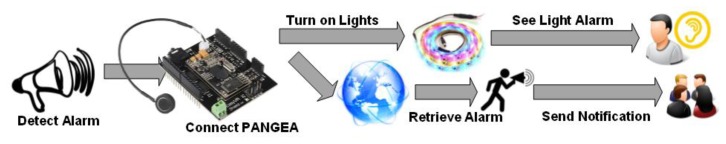
The integration of Arduino and voice recognition module in PANGEA.

The sensor agent controls the following hardware components, which can be seen in Figure 3: Arduino Duemilanove board, EasyVR voice recognition module and a set of 100 light-emitting diodes (LEDs) of various colors.

The speaker is measuring sounds from the environment when it detects a pattern similar to that of a siren. The microcontroller then activates and emits flickering lights using high visibility LEDs, which draw the user's attention. This is useful for the hearing-impaired, for example, who are unable to hear the sound of an alarm from a conventional acoustic device.

As shown in Figure 3, the sensor agent is connected to PANGEA by means of the IRC protocol. Thus, the organization created using PANGEA can receive and analyze the information received from the sensor agent. Based on this information, the organization can monitor the environment and define services, such as notification systems, to send alerts and notifications to users through different mechanisms. The information provided by sensor agents is used from an organizational point of view to analyze the functioning of the current organizations and to recommend possible re-organization solutions.

#### 3.3.3. Portable Sensor (TAG), VibratorAgent

One of the advantages of using wireless sensor networks (WSN) is the ability to wirelessly interconnect multiple electronic devices in a simple and scalable way. In general, the devices connected to such networks can be considered autonomous nodes, capable of running embedded programs, with a low battery consumption level and with a very simple logic of operation. This section presents the proposed solution whose main function is to notify the user about an alarm condition through the use of the sound recognition module described in Section 3.3.1

**Figure 4.**
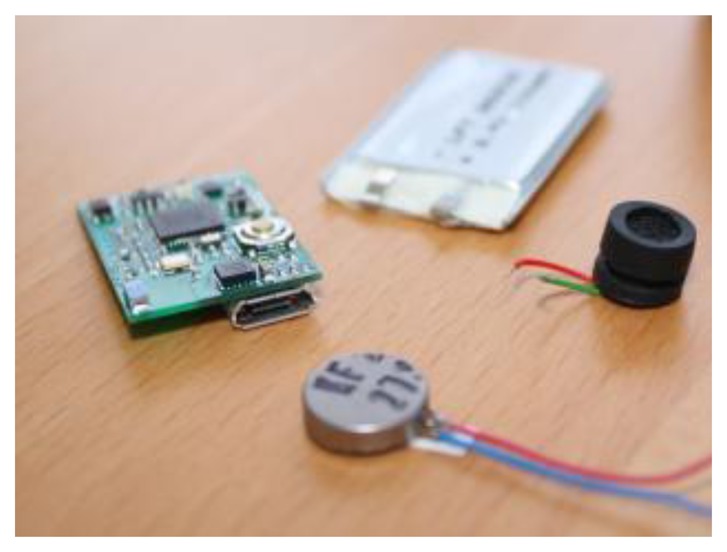
Image of the Arduino Mini Pro actual size.

To facilitate the portability and usability of the location device, we opted for a solution based on Arduino Nano Pro v3. It is a small sized microcontroller that the user can easily wear, since it can be placed in a bag or attached to clothes. In Figure 4, it is possible to see the actual size of the chosen device.

As stated above, the main goal of using this hardware in our proposal is to notify disabled users who, due to physical or mobility issues, cannot observe the emergency light or sound alerts that are generated in an emergency situation, but must respond to them nevertheless. The battery of the Arduino microcontroller has a voltage of 3.7 V and a capacity of 370 mAh, ensuring the correct operation of the device during 48 h. The interconnection of the TAG with the PANGEA platform is carried out using the TCP/IP protocol and the IRC message format type defined in RFC1459 [68]. It is also necessary to use shield-type WiFly RN-171 [69], which provides wireless communication with the central server where the main agents of the PANGEA platform reside.

The agent embedded in the TAG is continuously subscribed to the Alarm and Monitor virtual organization. When any of the predefined events is triggered, the event is communicated to the user in the form of vibrations (using the vibration sensor). The event code is transformed into vibrations using an SOS in Morse code. The following section explains the algorithms used in PANGEA for sending notifications to the nodes to the network.

#### 3.3.4. Sending Algorithms

**Figure 5.**
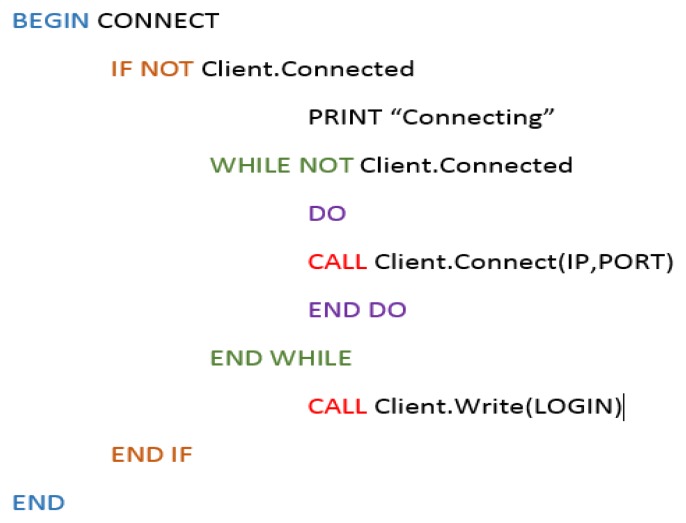
Simple connection agent to the PANGEA system.

The sensor agent executes specific algorithms to fulfill responsibilities inside the organization. It is important to take into account that the sensor agent plays a role inside the defined organization by means of the PANGEA platform. The code in Figure 5 shows how the sensor agent connects to the PANGEA platform and is executed only when the micro-controller has been initiated. Once inserted into the PANGEA platform, it joins the rest of the platform agents so that it can interact with them. After registering in the platform, the agent sends the information about the services it provides. That information is sent to the ServiceAgent, who can then provide it to other agents. The services will depend on the sensors connected to the Arduino device, and the agent deployed in the Arduino device will contain the information about the available sensors.

**Figure 6.**
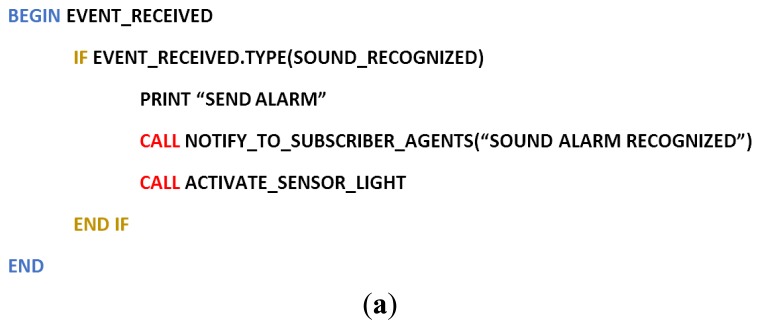

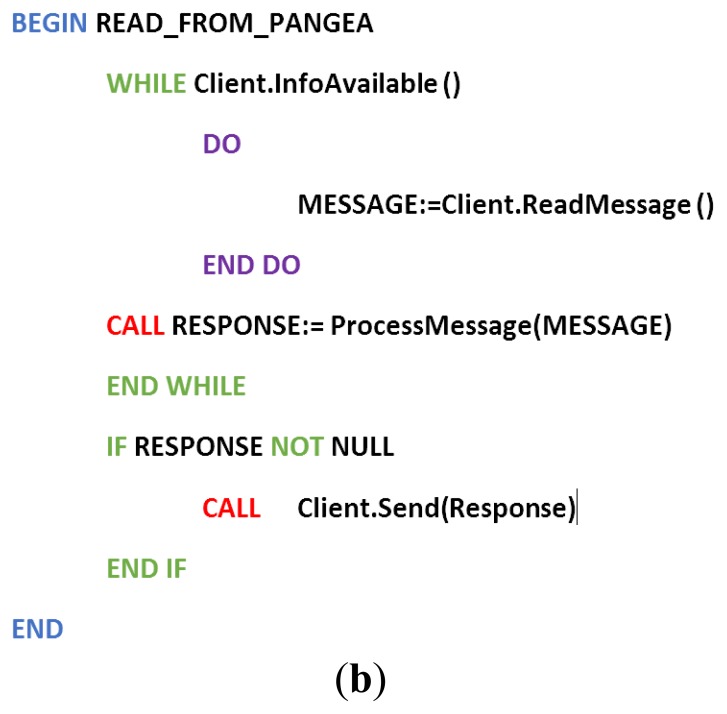
Treatment Pseudocode of a fire alarm event in PANGEA. (**a**) The agent receives an event and responds to it; (**b**) The receiver remains blocked from receiving messages.

The sensor agent then communicates with the organization using an algorithm in charge of confirming whether there are any requests from the PANGEA-based organization. The following image in Figure 6a shows how the alarm event is recognized and then how the fire event is treated in the system. The notifications are sent to the subscribed agent in the platform, after which the notifications are received by the subscriber. The subscriber remains blocked until it receives a message, as seen in Figure 6b.

Upon activating the microprocessor, the code is continuously executed.

When a known pattern is recognized, an event is initiated. In our example, the event is G1_ENCENDER_LUZ, which gives the order to turn on the bright LEDS and alert all of the agents connected to the PANGEA platform of the existing fire.

### 3.4. Detection and Tracking Movements by the Camera Agent

In addition to the sensor agent, another kind of sensor agent has been defined in our approach: a camera agent. The camera agent is dedicated to controlling an IP camera that tracks and detects movements. The camera is controlled by the camera agent, which is responsible for obtaining images and detecting movements. The wireless fidelity (WiFi) camera is activated remotely by an agent or automatically when the agent receives an alert through a sensor.

The processing system is very basic. The objective is to deploy an agent that manages the camera remotely; however, it must be able to interact with the camera as an embedded agent. The implementation is based on basic image processing techniques [70]. The process of motion detection and tracking consists of subtracting images and calculating the areas of the remaining objects to track specific surfaces. During the process of subtracting images, it is only necessary to consider two images captured without any movement of the camera between the two captures. The difference between the images is calculated using the red, green, blue (RGB) color channels. The image is transformed into a binary image, which takes on the value of one for a given pixel if that pixel exceeds the defined threshold for any of the channels.

Once the images have been captured, the subtraction of the images is calculated, and based on the differences, the detection of the remaining figures in the image is calculated. Using dilation and erosion algorithms [70,71], the images are then filled in order to avoid the presence of empty spaces. This is followed by the process of filling the remaining voids, applying an algorithm based on morphological reconstruction [72].

**Figure 7.**
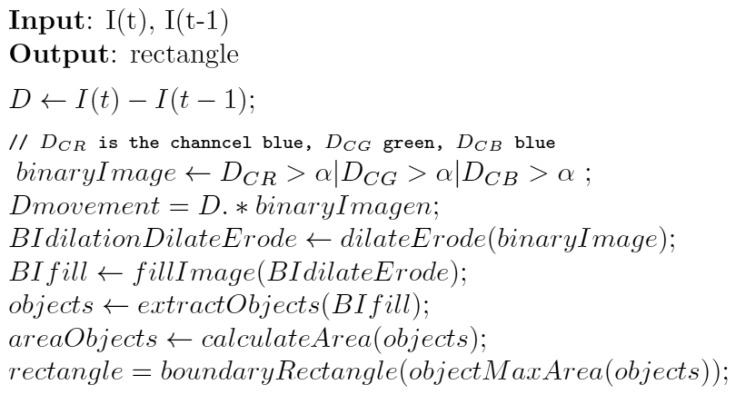
Movement detection.

To complete the process of filling the spaces, a binary image of each of the objects is obtained. This is a simple process by which each pixel with a value of one in the binary image is considered a new object if it does not contain an adjoining pixel with a value of one that was previously tagged as a new object. This is the process followed by MATLAB for object recognition [70]. Once the objects have been located, the area of each object is calculated, and the object identified as having the largest size is tracked. A summary of the motion detection process can be seen in Figure 7

In order to track a detected object, the objected is framed in a rectangle; the camera moves when the object exceeds the defined margins of the image that center the object in the camera. The camera moves a predetermined number of degrees, stops and captures another image to determine the position of the moving objects. It will move again if the object is found outside the central margins of the image. As the camera cannot detect the distance of the objects, it is unable to calculate the speed of movement for the object and, thus, uses this method of tracking.

### 3.5. Global Functioning of the System

**Figure 8.**
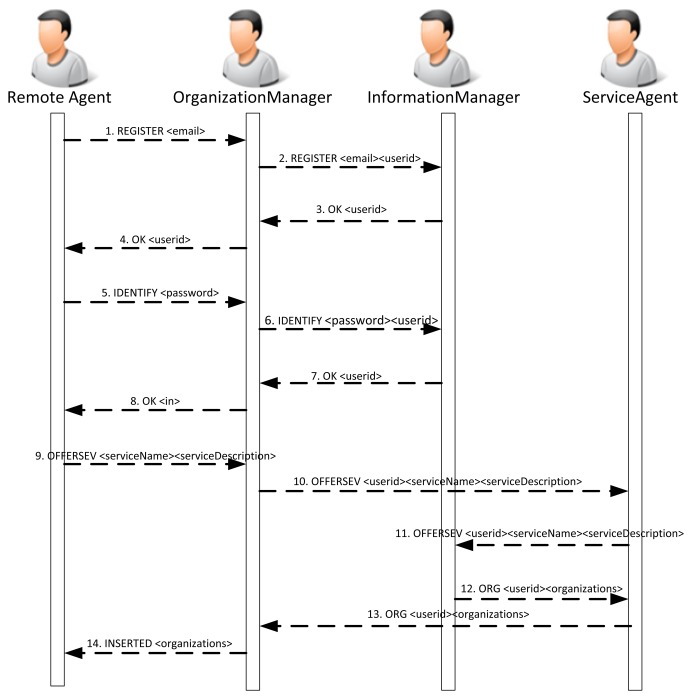
Sequence diagram describing the interaction between and remote agent and the platform during the registration process.

Generally speaking, the inclusion of agents that manage sensors in the platform can be done in two ways: for the camera agent and for the sensor agent. The camera cannot be embedded with an agent, since it has not been programmed this way. Instead, the agent is installed remotely and remotely accesses the web services that have been provided to implement its behavior. The agent actually behaves as if it were deployed on the camera, and it is responsible for accessing the functionality of the camera. A remote agent will generally behave according to Figure 8.

**Figure 9.**
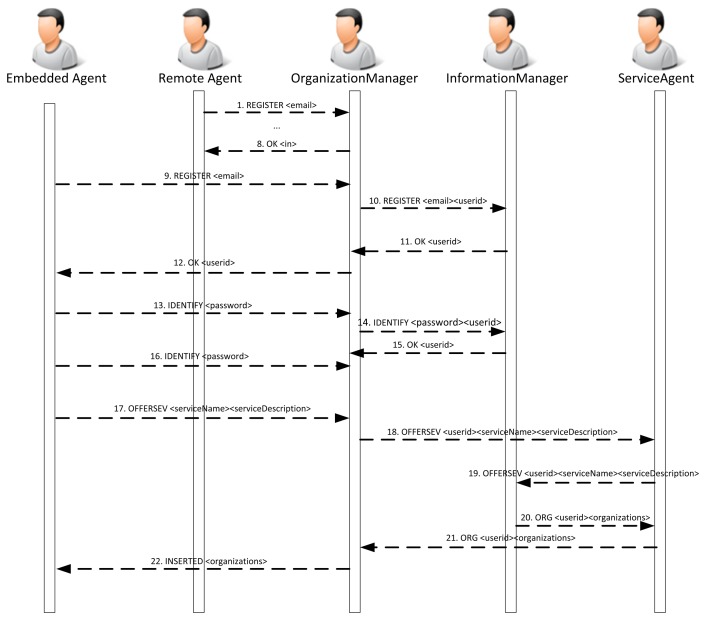
Sequence diagram describing the interaction among the remote agent, the embedded agent and the platform during the registration process.

If it is possible to embed part of the agent's behavior in the device, the services will be distributed between the embedded and the remote agent. In this case, the sequence diagram to register the agents and services is similar when there is only a remote agent. The remote agent may not be necessary if all of the services can be included in the embedded agent. Figure 9 shows the sequence of messages.

**Figure 10.**
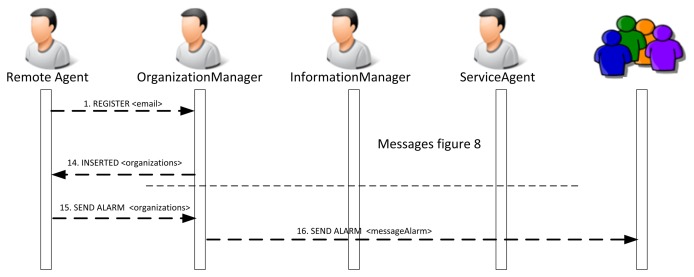
Sequence diagram describing the interaction between the sensor agent and the organization.

Figure 10 presents the sequence of exchanges between the PANGEA platform agents and a sensor agent when the sensor agent is registered in the platform. As can be seen in Figure 8, the sensor agent registers different services that can be used by the rest of the agents in the platform. It is represented in Message 9. More specifically, the sensor agent offers a notification service that sends information about sounds detected in the environment. The service is similar to a traditional news service subscription. An agent is registered and receives information from the detected sounds.

**Figure 11.**
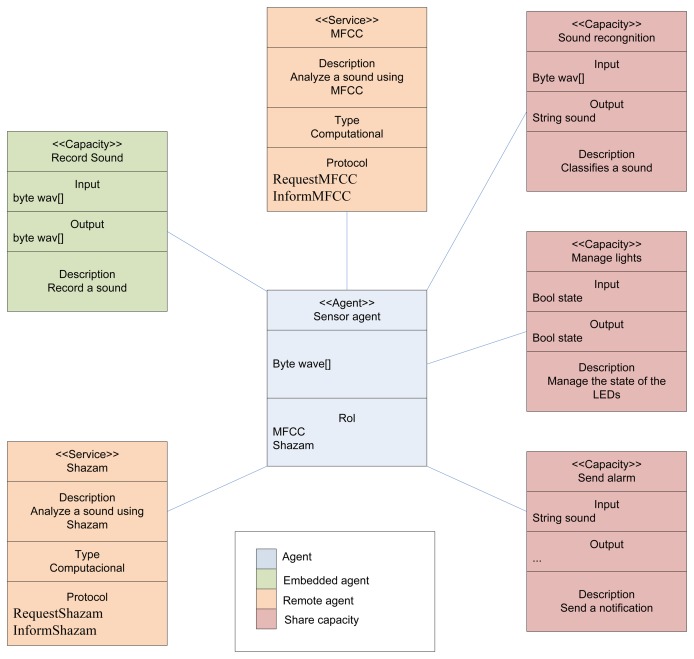
Class diagram of the sensor agent. MFCC, Mel frequency cepstral coefficients.

Figure 11 shows the list of capabilities and services provided by the embedded and the remote agent. Some of these skills and services are reflected in Figure 8.

The first time the sensor agent accesses the platform, a registration protocol is executed. The sensor agent is assigned a unique identifier. Then, an authentication process is executed, and the OrganizationManager agent sends a request (via Frame 6) to the information agent to validate the user data. Once the sensor agent is validated, a replicated agent is instantiated and it communicates with the OrganizationManager and the ServiceAgent agents to register the sound detection service.

The sensor agent is part of the organization; when a sound is detected, the agents subscribed to the notification service are notified.

## 4. Case Study: Developing an Intelligent Environment for Alarm Detection

The system was developed in a laboratory belonging to the research group. The laboratory includes different types of sensors, such as lighting, heating, IP cameras WiFi, sirens and others, all connected wirelessly through a wireless sensor network. With the exception of the IP camera, the remaining sensors are not connected to the system that was developed; this will make it possible to integrate the system into homes without needing to spend money connecting hardware, such as gas or motion detectors, to the sensor network. The sensors and the system are connected through a siren or bell. The system detects and classifies the different sounds using sound recognition modules that interpret the sounds and execute actions according to the agents that have been embedded into the devices. This allows the system to be integrated into the home without a significant increase in cost.

The following alarms were used during the case study: smoke detector, gas detector and robbery detector. The activation of these alarms affected the IP camera and the illuminated bands.

The room was equipped with an Arduino device. The Arduino is connected with a WiFi module. The agent is connected to the PANGEA platform, which is installed remotely in a mode server. The illuminated bands are connected to the Arduino board. The web camera is controlled by an agent located at the central server of the PANGEA platform, thus eliminating the need for having any equipment in the laboratory.

PANGEA will contain the agents from the Translator Organization, the Central Control Organization and the agents of the organizations belonging to the case study indicated in Section 3.1. If another case study were included, it would only be necessary to include the organizations with the agents belonging to the new case study; the Translator Organization and Central Control Organization would be shared with all of the case studies.

## 5. Results

In order to carry out the study, the system performance was analyzed to ensure that it correctly classified the sounds coming from the different alarms and that the alarms initiated the correct functioning of the IP camera. The PANGEA platform was installed on a PC containing an Intel Core 2 Duo P9700 processor with 2.8 GHz, connected to the network through a 100-Mbit connection. The web camera records in color with a resolution of 640 × 480 pixels. All agents are deployed on this computer, except the sensor agent and vibrator agent, which are located in the Arduino devices. It would be possible to run agents on other computers if so required, for example, because of either the processing or the number of suborganizations deployed to manage the WSNs.

In the first test, the robbery alarm activated the camera. When the alarm was activated, the sensor agent automatically alerted the agent responsible for supervising the camera, so that it could initiate the recording and begin sending photographs. During this time, the camera detects the movements and tracks people according to the orders indicated by the camera agent.

In order to test the operation of the gas and smoke sensors, the respective sound alarm was activated in order to ensure that the system was capable of recognizing the sounds without error. The alerts were sent to email, and the camera was activated according to its configuration. As a result of the activation of the gas and smoke alarms, the illuminated bands were activated to alert the user in case of any hearing impairment. The illuminated bands are connected to the Arduino device, which is in charge of activating a sequence of colors according to the type of alarm.

In order to ensure that the system correctly detects the sound, background music was played in the room prior to activating the alarm. In this case, it was noted that sound recognition could be affected if the volume of the music were very loud or if the music were close to the microphone. In order to avoid a situation in which a loud sound could muffle the sound of the sirens and not activate the alarms, we chose to activate a warning when the volume exceeded a predefined decibel level, thus ensuring that the alarm could not be drowned out by another sound.

**Figure 12.**
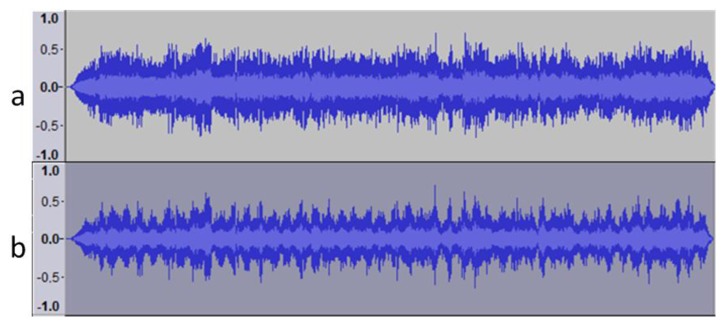
(**a**) Original sound; (**b**) Sound obtained after applying a fuzzy filter to the original sound.

In order to analyze the sound recognition, the original sound of the alarm was recorded. The sound spectrum of the alarm can be seen in Figure 12a. Figure 12b takes the base sound and applies a fuzzy distortion to modify the sound frequencies. It was possible to notice the difference when the new signal was played.

The proposed approach was evaluated using a dataset of 42 alarm sounds available at soundjax [73]. The number of sounds is higher than the number of sounds supported by EasyVR; thus, it will be necessary to send sound to the replicated sensor agent to recognize unclassified sounds. The average length for the sounds is 2.83 s, and the deviation is 2.93 s. Different alarms were selected from the dataset, and the detection was tested using the following algorithms: EasyVR, MFCC and Shazam.

The parameters settings for the MFCC algorithm were determined as indicated in [66]. The frame window was established at around 25 ms. The overlap is 50%, and the number of cepstral coefficients is 12 for each of the fingerprints. For each of the alarms, we defined several cepstral coefficient vectors that depend on the frame window and overlap. The frame window was extended to 200 ms to obtain the fingerprints. Different classifiers were used to determine the sounds [74,75], more specifically SVM, Bayes Net and J48 were used.

Different tests were executed, increasing the frame window size in increments of 25 until reaching 200 s and varying the classifiers. A 10-fold cross-validation was applied, and the best results obtained were for a 200-second frame window and the Bayes Net classifier. The set up for the EasyVR algorithm used the default parameters, while the Shazam algorithm used a landmark density of 400 per second and 600 per recognition. These values were selected due to the short length of the sounds.

**Figure 13.**
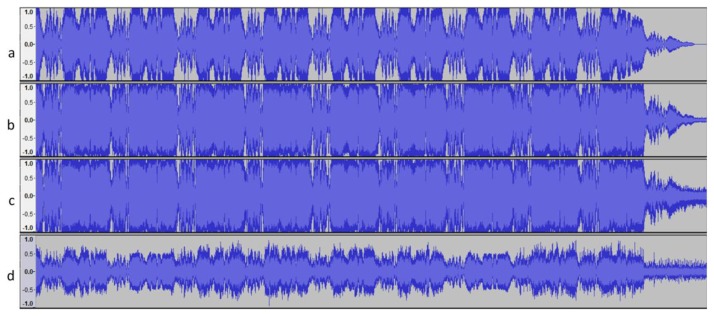
(**a**) Original sound; (**b**) Sound with white noise; (**c**) Gaussian noise; (**d**) Original sound randomly merged with an existing alarm.

Different noises were introduced to evaluate the system: white noise, 20% of the sound; Gaussian noise with a mean of zero and a standard deviation of half the interval of maximum variation. Additionally, 42 sounds were created by merging existing sounds. Figure 13 shows the results obtained before and after the introduction of the alterations.

The sounds were played close to the Arduino device. The behavior of the sensor agent is shown in the Table 1:

The embedded and remote agents can analyze the different sound and send the alerts to the agents in the organization, thus allowing the camera agent to react to the events. Furthermore, the embedded and remote agent act in the same manner for the system, which allows the services with low processing that are carried out in the embedded agent to be separated transparently from the services with high processing that are executed in the remote agent.

**Figure 14.**
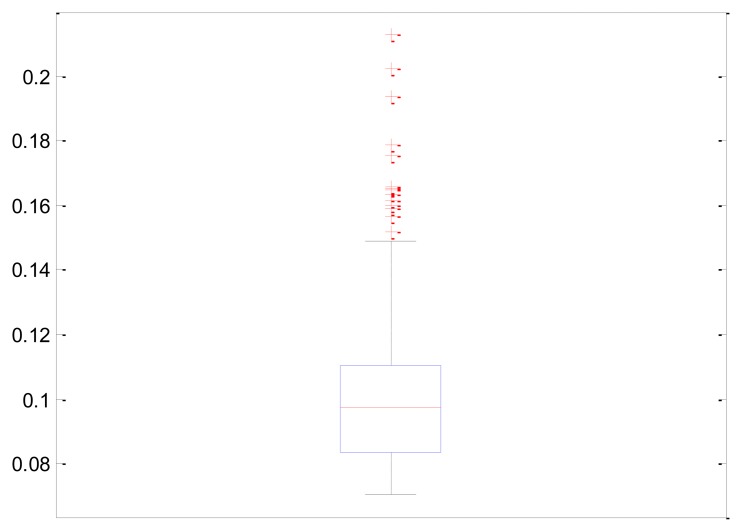
Box plot with the information about the execution time during the processing.

In order to analyze the system performance, a study was performed on the number of images per second that the system was capable of processing. To do so, an intensive processing was performed in which a sequence of 1001 images was passed through the algorithm, and the response times were analyzed. The total time for the analysis was 98.075 s. The average execution time was 0.098075 with a deviation of 0.019300. The time distribution is shown in Figure 14.

The average size of the images was 15.7 Kb, which takes into consideration that the system processes 10.19 images per second, and the bandwidth necessary to process all of the information is 1280.65 Kb. As the bandwidth could be a bit greater for some ADLS (Asymmetric Digital Subscriber Line) connections, the system can establish the number of images processed per second.

## 6. Conclusions

The PANGEA platform facilitates the development of ambient intelligence systems. The platform can connect different devices, thus facilitating the integration of the information and the decisions made based on the information provided by the sensors. The architecture can carry out complex processes that cannot be done with low-processing hardware, such as Arduino, or with devices that lack processing capabilities, such as an IP camera.

One of the main novelties of the system is the incorporation of agents in Arduino devices, which permits the simple incorporation of different sensors and actuators onto the PANGEA platform.

The combination of Arduino and the voice recognition module allows the recognition of different sounds for which different responses can be programmed. This characteristic is quite relevant, since it makes it possible to incorporate different devices, such as bells and smoke, robbery and gas alarms, into the system using sound recognition. This system reduces costs, since it can incorporate any device that sounds an alarm. Due to the recognition of alarms, it is possible to initiate different actions, such as activating illuminated bands, sending vibrator movements to a user with a vibrator sensor, sending alert messages or activating an IP camera to remotely track an incident.

One of the main advantages of this system is the low cost of installation, since it is not necessary to have a computer that is turned on; low cost hardware is also used. The greatest associated cost is an Internet connection, which is necessary to supervise the environment.

## Figures and Tables

**Table 1.** Audio recognition.

	Original			White Noise			Gaussian Noise			Mixture		
			
	Success	Fail	Ignored	Success	Fail	Ignored	Success	Fail	Ignored	Success	Fail	Ignored
EasyVR	32	0	10	25	0	17	21	0	21	2	1	39
MFCC	36	0	6	30	1	11	27	2	13	17	5	62
Shazam	42	0	0	41	0	1	38	3	1	63	21	0

## References

[1] Fernández J., Campillo P., Fuentes R., Pavón J. (2014). Opportunistic control mechanisms for ambience intelligence worlds. Expert Syst. Appl. Int. J..

[2] Jayaputera G.T., Zaslavsky A., Loke S.W. (2007). Enabling run-time composition and support for heterogeneous pervasive multi-agent systems. J. Syst. Softw..

[3] Gómez-Romero J., Serrano M.A., Patricio M.A., García J., Molina J.M. (2012). Context-based scene recognition from visual data in smart homes: An Information Fusion approach. J. Pers. Ubiquitous Comput..

[4] Gast M., Gast M.S. (2002). 802.11 Wireless Networks: The Definitive Guide.

[5] Bajo J., Corchado J.M., de Paz Y., de Paz J.F., Rodríguez S., Martin Q., Abraham A. (2009). SHOMAS: Intelligent Guidance and Suggestions in Shopping Centres. Appl. Soft Comput..

[6] Fonseca S.P., Martin P., Griss L., Letsinger R. (2001). An Agent Mediated E-Commerce Environment for the Mobile Shopper, Hewlett-Packard Laboratories.

[7] Kowalczyk R., Ulieru M., Unland R. (2002). Integrating Mobile and Intelligent Agents in Advanced e-Commerce: A Survey.

[8] Santofimia M.J., Fahlman S.E., del Toro X., Moya F., Lopez J.C. (2011). A semantic model for actions and events in ambient intelligence. Eng. Appl. Artif. Intell..

[9] Guivarch V., Camps V., Péninou A. (2013). AMADEUS: An adaptive multi-agent system to learn a user's recurring actions in ambient systems. Adv. Distrib. Comput. Artif. Intell. J..

[10] Venturini V., Carbo J., Molina J.M. (2012). Methodological design and comparative evaluation of a MAS providing AmI. Expert Syst. Appl. Int. J..

[11] Tapia D.I., Alonso S., García O., Corchado J.M., Bajo J. Wireless sensor networks, real-time locating systems and multi-agent systems: The perfect team.

[12] Delgado-Roman M.C., Sierra C. (2013). A Multi-agent Approach to Energy-Aware Wireless Sensor Networks Organization. Lecture Notes Comput. Sci..

[13] Ansola P.G., de las Morenas J., García A., Otamendi J. (2013). Distributed decision support system for airport ground handling management using WSN and MAS. Eng. Appl. Artif. Intell..

[14] Delgado-Roman M.C., Pujol-Gonzale M., Sierra C. (2013). Multi-Agent Co-Ordination of Wireless Sensor Networks. Lecture Notes Comput. Sci..

[15] Barret S.F. (2010). Arduino Microcontroller Processing for Everyone!.

[16] Zato C., Villarrubia G., Sánchez A., Barri I., Rubión E., Fernández A., Rebate C., Cabo J.A., Álamos T., Sanz J., Seco J.J, Bajo J., Corchado J.M. (2012). PANGEA—Platform for Automatic Construction of Organizations of Intelligent Agents.

[17] Acampora G., Loia V. (2008). A proposal of ubiquitous fuzzy computing for Ambient Intelligence. Inf. Sci..

[18] Want R., Hopper A., Falcao V., Gibbons J. (1992). The Active Badge Location System. ACM Trans. Inf. Syst..

[19] Park D., Hwang S., Kim A., Chang B. A Context-Aware Smart Tourist Guide Application for an Old Palace.

[20] Skov M., Hoegh R. (2006). Supporting information access in a hospital ward by a context-aware mobile electronic patient record. J. Perv. Ubiquitous Comput..

[21] Dey A.K. Context-aware computing: The CyberDesk project.

[22] Abdaoui A., El-Fouly T.M. (2014). TOSSIM and distributed binary consensus algorithm in wireless sensor networks. J. Netw. Comput. Appl..

[23] Levis P., Culler D. Maté: A tiny virtual machine for sensor networks, ACM SIGARCH Computer Architecture News-Special Issue.

[24] Fok C., Roman G., Lu C. Mobile agent middleware for sensor networks: An application case study.

[25] Molla M.M., Ahamed S.I. A Survey of Middleware for Sensor Network and Challenges.

[26] Lewis P.L., Welsh N., Culler M.D. TOSSIM: Accurate and scalable simulation of entire TinyOS applications.

[27] Lim C.H., Anthony P., Fan L.C. (2009). Applying multi-agent system in a context aware. Borneo Sci..

[28] Uhm Y., Hwang Z., Lee M., Kim Y., Kim G., Park S. A Context-Aware Multi-Agent System for Building Intelligent Services by the Classification of Rule and Ontology in a Smart Home.

[29] Kaluža B., Luštrek M., Dovgan E., Gams M. Context-aware MAS to support elderly people (demonstration).

[30] Ning K., Yang R. (2006). MAS based embedded control system design method and a robot development paradigm. Mechatronics.

[31] Doctor F., Hagras H., Callaghan V. (2005). A type-2 fuzzy embedded agent to realise ambient intelligence in ubiquitous computing environments. Inf. Sci..

[32] Agüero J., Rebollo M., Carrascosa C., Julián V. Towards on embedded agent model for Android mobiles.

[33] Su C.-J., Wu C.-Y. (2011). JADE implemented mobile multi-agent based, distributed information platform for pervasive health care monitoring. Appl. Soft Comput..

[34] Agüero J., Rebollo M., Carrascosa C., Julián V. (2010). Model-driven development for ubiquitous MAS. Ambient Intelligence and Future Trends-International Symposium on Ambient Intelligence.

[35] Purusothaman S.R.R.D., Rajesh R., Bajaj K.K., Vijayaraghavan V. Implementation of Arduino-based multi-agent system for rural Indian microgrids.

[36] Santi A., Guidi M., Ricci A. JaCa-Android: An agent-based platform for building smart mobile applications.

[37] Staneva D., Gacheva P. CompSysTech'2004 the Communication Infrastructure of the MAPNET Mobile-Agent Platform.

[38] Milojicic D., Breugst M., Busse I., Campbell J., Covaci S., Friedman B., Kosaka K., Lange D., Ono K., Oshima M., Tham C., Virdhagriswaran S., White J. (1998). MASIF: The OMG mobile agent system interoperability facility. Pers. Technol..

[39] Waligóra I., Małysiak-Mrozek B., Mrozek D. (2010). Uniwersalna platforma wieloagentowa UMAP. Stud. Inf..

[40] Gascueña J.M., Navarro E., Fernández-Caballero A., Pavón J. Development of a Code Generator for the ICARO Agent Framework. Advances in Artificial Intelligence–IBERAMIA 2012.

[41] Kira Z., Schultz A.C. Continuous and Embedded Learning for Multi-Agent Systems.

[42] Raspberry Pi. http://raspberrypi.org.

[43] Li J., Huai J., Hu C., Zhu Y. (2010). A secure collaboration service for dynamic virtual organizations. Inf. Sci..

[44] Ahonen H., de Alvarenga A.G., Provedel A. (2009). Selection and scheduling in a virtual organisation environment with a service broker. Comput. Ind. Eng..

[45] Zato C., de Paz J.F., de Luis D., Bajo J., Corchado J.M. (2012). Model for assigning roles automatically in egovernment virtual organizations. Expert Syst. Appl..

[46] van Do T. (2010). Modeling a resource contention in the management of virtual organizations. Inf. Sci..

[47] Zhang G., Jiang J., Su Z., Qi M., Fang H. (2010). Searching for overlapping coalitions in multiple virtual organizations. Inf. Sci..

[48] Cossentino M., Gaud N., Hilaire V., Galland S., Koukam A. (2010). ASPECS: An Agent-Oriented Software Process for Engineering Complex Systems - How to Design Agent Societies Under a Holonic Perspective. J. Auton. Agents Multi-Agent Syst. (JAAMAS).

[49] Gutknecht O., Ferber J. (2000). The MADKIT Agent Architecture. Lecture Notes Comput. Sci..

[50] Patel J., Luke W.T., Jennings N.R., Luck M. (2005). Agent-Based Virtual Organisations for the Grid. Int. J. Multi-Agent Grid Syst..

[51] Tapia D.I., Fraile J.A., Rodríguez S., Alonso R.S., Corchado J.M. (2013). Integrating hardware agents into an enhanced multi-agent architecture for Ambient Intelligence systems. Inf. Sci..

[52] Fernández J.M., Fuentes R., Pavón J. (2011). A Dynamic Context-Aware Architecture for Ambient Intelligence (IWANN). Adv. Comput. Intell..

[53] Ferber J., Gutknecht O., Michel F. From Agents to Organizations: an Organizational View of Multi-Agent Systems.

[54] Argente E., Giret A., Valero S., Julian V., Botti V. Survey of MAS Methods and Platforms focusing on organizational concepts.

[55] Zato C., Rodríguez S., Tapia D., Corchado J.M., Bajo J. Virtual Organizations of agents for monitoring elderly and disabled people in geriatric residences.

[56] Zato C., Rodríguez S., Sánchez A., Villarrubia G., Bajo J., Corchado J.M. (2013). Personalization of the Workplace through a Proximity Detection System Using User Profiles. Int. J. Distrib. Sens. Netw..

[57] Zato C., Villarrubia G., Sánchez A., Bajo J., Corchado J.M. (2013). PANGEA: A New Platform for Developing Virtual Organizations of Agents. Int. J. Artif. Intell. (IJAI).

[58] Web PANGEA. http://pangea.usal.es/.

[59] Fuentes-Fernandez R., Gomez-Sanz J.J., Pavon J. (2007). Model integration in agent-oriented development. Int. J. Agent-Oriented Softw. Eng..

[60] Arduino Website. http://www.arduino.cc/.

[61] Propeller Microcontroller Website. http://www.parallax.com/propeller/.

[62] BeagleBoard Website. http://beagleboard.org/.

[63] EasyVR Voice Recognition Module. https://www.sparkfun.com/products/10685.

[64] Universal Asynchronous Receiver/Transmitter. http://en.wikipedia.org/wiki/Universal_asynchronous_receiver/transmitter.

[65] Wang A.L.-C. An Industrial-Strength Audio Search Algorithm.

[66] Jothilakshmi S., Ramalingam V., Palanivel S. (2009). Unsupervised speaker segmentation with residual phase and MFCC features. Expert Syst. Appl..

[67] Kaminskas M., Ricci F. (2012). Contextual music information retrieval and recommendation: State of the art and challenges. Comput. Sci. Rev..

[68] Internet Relay Chat Protocol RFC1459. http://tools.ietf.org/html/rfc1459.html.

[69] RN171 module. http://www.microchip.com/wwwproducts/Devices.aspx?product=RN171.

[70] Gonzalez R.C., Woods R.E., Eddins S.L. (2009). Digital Image Processing Using MATLAB.

[71] van den Boomgard R., van Balen R. (1992). Methods for Fast Morphological Image Transforms Using Bitmapped Images. Comput. Vision Gr. Image Process. Gr. Models Image Process..

[72] Soille P. (1999). Morphological Image Analysis: Principles and Applications.

[73] SoundJax. http://soundjax.com/alarm-1.html.

[74] Wang S., Tang Z., Li S. (2011). Design and Implementation of an Audio Classification System Based on SVM. Procedia Eng..

[75] Xie C., Cao X., He L. (2012). Algorithm of Abnormal Audio Recognition Based on Improved MFCC. Procedia Eng..

